# Estimating the neural spike train from an unfused tetanic signal of low-threshold motor units using convolutive blind source separation

**DOI:** 10.1186/s12938-023-01076-0

**Published:** 2023-02-07

**Authors:** Robin Rohlén, Jonathan Lundsberg, Christian Antfolk

**Affiliations:** 1 Department of Biomedical Engineering, Lund University, 221 00 Lund, Sweden; 2 Department of Radiation Sciences, Biomedical Engineering, Radiation Physics, Umeå University, Umeå, Sweden

**Keywords:** Convolutive blind source separation, Spike train, Unfused tetanus, Twitch, Motor unit, Motoneuron

## Abstract

**Background:**

Individual motor units have been imaged using ultrafast ultrasound based on separating ultrasound images into motor unit twitches (unfused tetanus) evoked by the motoneuronal spike train. Currently, the spike train is estimated from the unfused tetanic signal using a Haar wavelet method (HWM). Although this ultrasound technique has great potential to provide comprehensive access to the neural drive to muscles for a large population of motor units simultaneously, the method has a limited identification rate of the active motor units. The estimation of spikes partly explains the limitation. Since the HWM may be sensitive to noise and unfused tetanic signals often are noisy, we must consider alternative methods with at least similar performance and robust against noise, among other factors.

**Results:**

This study aimed to estimate spike trains from simulated and experimental unfused tetani using a convolutive blind source separation (CBSS) algorithm and compare it against HWM. We evaluated the parameters of CBSS using simulations and compared the performance of CBSS against the HWM using simulated and experimental unfused tetanic signals from voluntary contractions of humans and evoked contraction of rats. We found that CBSS had a higher performance than HWM with respect to the simulated firings than HWM (97.5 ± 2.7 vs 96.9 ± 3.3, *p* < 0.001). In addition, we found that the estimated spike trains from CBSS and HWM highly agreed with the experimental spike trains (98.0% and 96.4%).

**Conclusions:**

This result implies that CBSS can be used to estimate the spike train of an unfused tetanic signal and can be used directly within the current ultrasound-based motor unit identification pipeline. Extending this approach to decomposing ultrasound images into spike trains directly is promising. However, it remains to be investigated in future studies where spatial information is inevitable as a discriminating factor.

**Supplementary Information:**

The online version contains supplementary material available at 10.1186/s12938-023-01076-0.

## Introduction

The smallest functional element that can be voluntarily activated in the neuromuscular systems comprises a motoneuron and a group of muscle fibres innervated by the motoneuron. This functional element is called a motor unit (MU). The gold standard for measuring the characteristics of a large population of MUs is based on high-density surface electromyography (sEMG) [1]. High-density sEMG consists of a grid of electrodes that records mixed MU activities superficially from the skin (< 20 mm). Then, the activity is decomposed into single MU spike trains by blind source separation (BSS) techniques, which use many electrodes [2, 3]. This approach provides access to the neural drive of the spinal cord via the motoneurons to specific muscles [4]. Although sEMG has been used successfully for MU analysis, it is well-known that it has limited spatial selectivity and field of view.

**Fig. 1:**
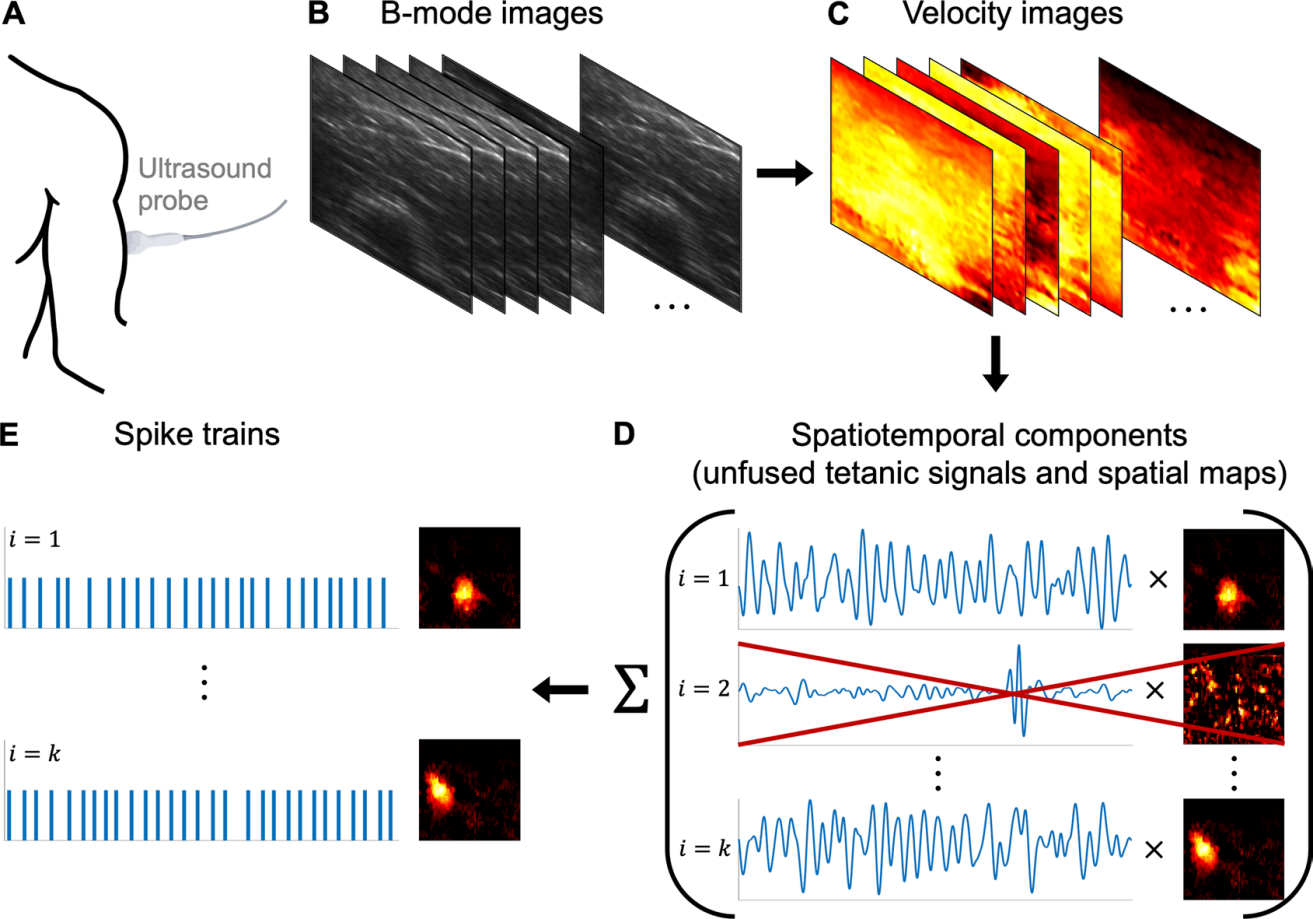
The methodological pipeline to identify voluntarily activated motor units (MUs) using ultrafast ultrasound. **A** The ultrasound probe is placed on the skin to record data from the muscle’s cross-section (transverse view). **B** The recorded B-mode images. **C** Calculating displacement velocity images. **D** Separating the velocity images into spatiotemporal components using *instantaneous* blind source separation (BSS) with a focus on spatial sparsity, where each component is associated with a time signal and a spatial image. A subset of the components is putative estimates of the (1) MU territory in the spatial maps and (2) a sequence of MU twitches (unfused tetanic signal) evoked by the spike trains. **E** The spike trains are estimated based on unfused tetani. Previously using a Haar wavelet method (HWM). In this study, we estimate the spikes trains in **E** using the unfused tetanic signals from **D** using convolutive blind source separation (CBSS) and compare the performance against another method, i.e. the Haar wavelet method (HWM)

Ultrafast ultrasound has been shown to image and analyse voluntarily activated MUs for a large field of view in the muscle (40 × 40 mm) [5–11], providing spatiotemporal mechanics at a high resolution (< 1 mm and > 1 kHz). This technique is based on recording radio frequency signals (B-mode images) (Fig. 1A, B), calculating displacement velocity images (Fig. 1C) and separating the images into spatiotemporal components, i.e. each component is associated with a (1) spatial map and (2) a time signal (Fig. 1D). A subset of the components is putative estimates of the (1) MU territory and (2) a sequence of MU twitches (unfused tetanic signal) evoked by the spike trains [5, 6, 9, 10]. The spike trains are estimated from the unfused tetanic signal (Fig. 1E). The spike train estimation is currently based on a Haar wavelet method (HWM), which detects the twitch onsets due to the large gradient changes, and it has good performance [12]. Although this ultrasound-based pipeline (Fig. 1) has great potential to provide comprehensive access to the neural drive of the spinal cord to muscles for a large population of MUs simultaneously, the method currently has a limited identification rate of the active MUs [6].

The estimation of spikes partly explains the current pipeline’s limited identification rate of active MUs from each estimated unfused tetanic signal that often is noisy [6, 13]. Since HWM is based on large gradients, the noise in the unfused tetanic signals may induce false positives. Therefore, we must consider alternative methods with at least similar performance and robust against noise, among other factors. An alternative method with great potential is based on convolutive blind source separation (CBSS), which has been used with great success on *multichannel* sEMG signals [2, 3, 14]. Since CBSS uses multiple samples over time to estimate each spike, it should be able to estimate spike trains from *single-channel* unfused tetanic signals robustly with high performance.

The main challenge of using CBSS to estimate the spike train from an unfused tetanic signal is that the unfused tetanus of a MU will consist of *variable* successive twitches [15–17], where the twitch duration is *longer* than the time between two succeeding spikes (i.e. an ISI). In contrast, the MU action potential is more *consistent*, and its duration is *shorter* than the ISI. Previous studies of spike train estimation of an unfused tetanic signal, i.e. the HWM, have exploited the similarity of each twitch rise (onset gradient) and observed that the twitch rise duration is shorter than the ISI [12]. Assuming the twitch rise is the action potential counterpart, and the remaining twitch activity is another additive noise component, we hypothesise that estimating the spike train using CBSS should have a high agreement with the ground truth. If this is the case, this method can be used directly in the current ultrasound-based pipeline to assess the neural drive to the muscle (Fig. 1). Indeed, it also implies the feasibility of extending the method to spatiotemporal data such that spikes can be estimated directly from the ultrasound-based image sequences.

This study aimed to estimate spike trains from simulated and experimental unfused tetani using a CBSS algorithm and compare it against the previously optimised and evaluated HWM [12]. We implemented the CBSS algorithm requiring three parameters based on fixed-point iterations [2, 3, 14] and peak detection [12]. We evaluated the parameters of CBSS using the estimated spike trains’ rate of agreement with the ground truth for the simulations. We also explored the algorithm’s parameters’ relation with the spike delta (time difference between truth or reference spikes and the estimated spikes) and its variation. Finally, we compared CBSS against the HWM based on their rate of agreement performance using simulated and experimental unfused tetanic signals from voluntary contractions of humans and evoked contraction of rats.

## Methods

The overview of the methods includes (1) simulations describing the model and the parameters, (2) the experimental data, (3) the CBSS algorithm to estimate the spike train from an unfused tetanic signal, (4) the parameter evaluation of CBSS using simulated data, and finally, (4) the comparison between CBSS and HWM using simulated and experimental data.

### Simulations

#### Simulation model

We used a simulation model that generated a motor unit (MU) unfused tetanus $Y\left(t\right)$ at time $t\ge 0$:

1 $$Y\left(t\right)=\sum_{i=1}^{n}{F}_{i}\left(t-{\Delta }_{i}\right)+\varepsilon \left(t\right),$$

where $t=\{\mathrm{0,1},\dots ,T\}$ is the time, and $n$ is the number of times that the MU fires. ${F}_{i}$ is the $i$th twitch generated by the MU at its $i$th spike at time instant ${\Delta }_{i}$. The duration of ${F}_{i}$ is longer than the inter-spike interval (ISI) ${\Delta }_{i+1}-{\Delta }_{i}$. $\varepsilon (t)$ is additive noise. Note that the twitch shape commonly varies within a contraction, which has been explained in other studies for voluntary contractions [15–17]. We express the corresponding spike train $s\left(t\right)\ge 0$ as:

2 $$s\left(t\right)=\sum_{i=1}^{n}\delta \left(t-{\Delta }_{i}\right),$$

where $\delta$ is the Dirac Delta function.

#### Simulation parameters

**Fig. 2:**
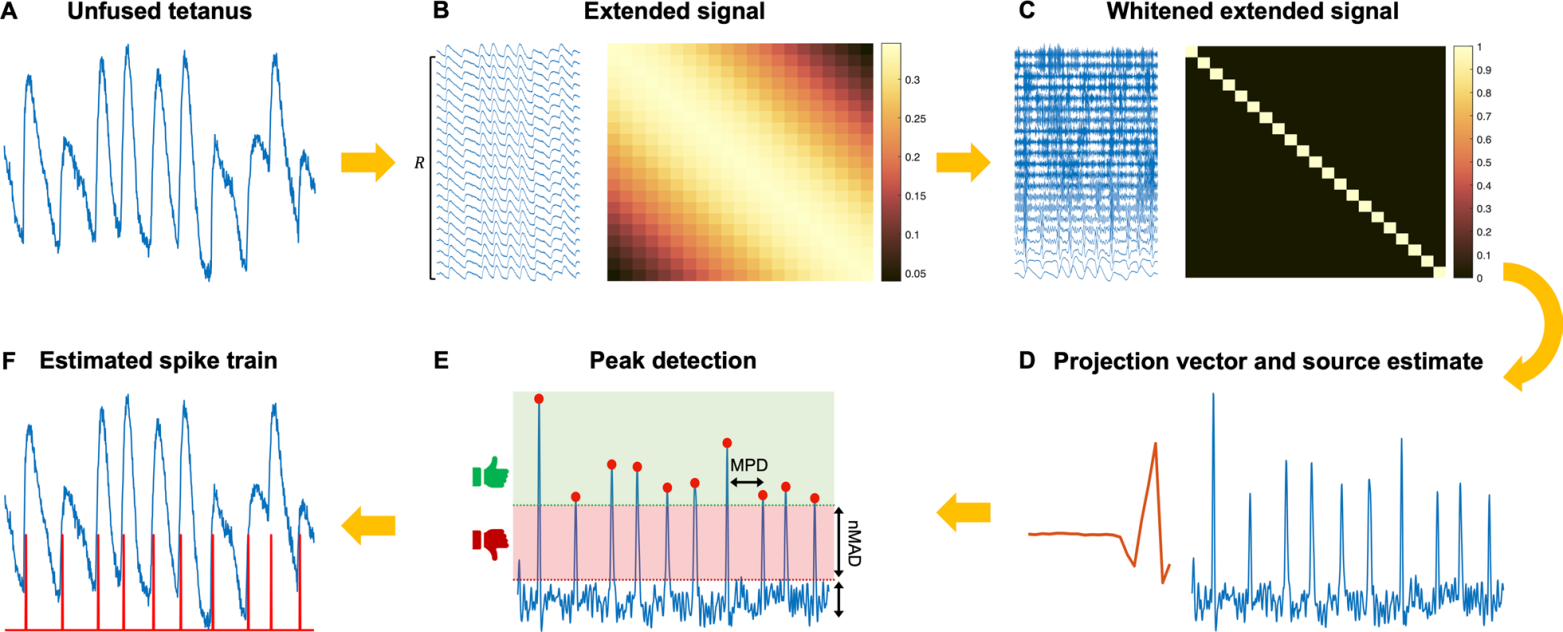
The different steps of the convolutive blind source separation algorithm. **A** The input signal is unfused tetanus. **B** The unfused tetanus is extended using the extension factor $R$, where the extended signal has a dense covariance matrix. **C** Performing whitening such that the covariance matrix equals the identity matrix. **D** The fixed-point algorithms find the separation (projection) vector (red line), and by projecting it on the whitened extended signal in **C**, the resulting output is the source estimate (blue line). **E** The time instants of the local maxima of the source estimate were detected using peak detection with two parameters: the minimal peak distance ($\mathrm{MPD}$) and the number of mean absolute deviations ($n\mathrm{MAD}$). **F** The time instants of the local maxima are the estimated firing times of the spike train ${\varvec{s}}$ (red line)

Similar to a previous study [12], each simulated unfused tetanus $Y\left(t\right)$ was based on simulating $n$ twitches and $n$ spikes (Fig. 2). The twitches ${F}_{i},i\in \{\mathrm{1,2},\dots ,n\}$ were simulated using a twitch model [17] with five parameters (Fig. 2A) that were randomly sampled to generate varying twitch-shapes (Fig. 2B) suitable for low-threshold MUs at low force levels [13] (see Additional file 1: Table S1). The $n$ spike times $\Delta =({\Delta }_{1},{\Delta }_{2},\dots ,{\Delta }_{n})$ were simulated using a Gaussian renewal process such that ${\Delta }_{i+1}-{\Delta }_{i} \sim N({\mu }_{ISI},\mathrm{CV})$, where ${\mu }_{ISI}$ and $\mathrm{CV}=\frac{{\sigma }_{ISI}}{{\mu }_{ISI}}$ is the mean ISI and coefficient of variation [18] (Fig. 2C). We simulated combinations of different average firing rates (8, 12, and 16 Hz) [6, 19] and ISI CV values (5, 20, and 40%) [20] feasible for low-threshold MUs.

Each twitch was summed to the respective spike resulting in an unfused tetanic signal (Fig. 2C). The unfused tetanus was *differentiated with respect to time* (from force to yank or displacement to velocity) [21] because of the stability of its mean value and velocity is used in MU identification using ultrasound. The last step was adding normally distributed noise so that the signal-to-noise ratio (SNR) was at three levels, i.e. 10, 20, and 30 dB. For more information on the simulations, see Additional file 1.

### Experimental signals

We retrospectively included, from previous studies, two datasets, including *n* = 21 unfused tetani [12]. The first dataset included three 2-s-long unfused tetani from the biceps brachii of three healthy human subjects (28.3 ± 0.6 years; one male and two females) at very low isometric force levels using the ultrasound-based MU identification pipeline with a (concentric) needle EMG electrode as [6] (see Additional file 1). The ultrasound and EMG systems were synchronised (sampling rates 2 kHz and 64 kHz).

The second dataset included 18 unfused tetani from the medial gastrocnemius of five adult female Wistar rats [22]. After a surgical procedure resulting in functionally isolated MUs, the Achilles tendon was connected to a force transducer while stretched to achieve isometric conditions where the force was measured simultaneously as a wire electrode (sampling rates 1 kHz and 10 kHz). Individual axons were stimulated based on average ISI times: 60, 70, 80, and 100 ms. For two rats, the last three stimuli intervals were used. The intervals between the individual stimuli were randomly set (mean ISI ± 50%). Before further analysis, the force-based signals were (1) filtered using a sixth-order zero-phase Butterworth bandpass filter with a high- and low-pass cut-off equal to 3 and 100 Hz and (2) differentiated. For detailed information about the functional isolation of MUs, see Additional file 1.

### The convolutive blind source separation algorithm to estimate the spike train

Previously a shift-invariant model has been used to describe a multichannel sEMG signal assuming (1) constant action potential waveforms for the same MU in one channel and (2) that the waveform duration is shorter than every ISI [2, 3, 14]. This study considers a single-channel unfused tetanus $Y\left(t\right)$ $,$ which has the following two characteristics: (1) the twitch waveforms for the same MU vary [15–17], and (2) the twitch waveform duration is longer than the ISI [16, 23]. Given this, we express the time derivative of the unfused tetanic signal as:

3 $$\widetilde{Y}\left(t\right)=\sum_{i=1}^{n}\sum_{l=1}^{L}{h}_{i}\left(l\right)s\left(t-l\right)+\widetilde{\varepsilon }\left(t\right),$$

where $i=\{1,\dots ,n\}$ denotes the $i\mathrm{th}$ spike, ${h}_{i}\left(l\right)$ is a twitch rise waveform of length $L$ as a response to the $i$th firing, $s\left(t\right)$ is the spike train at time $t$, and $\widetilde{\varepsilon }(t)$ is additive noise including the remaining signal of the twitch waveforms after subtracting the twitch rise waveform. The convolutive mixture with finite impulse response filters in Eq. (3) can be represented as a linear and an *instantaneous* mixture by extending the observation signal, source signal, and noise using their $R$ delayed versions [2, 3, 14]:

$$\widetilde{{\varvec{Y}}}\left(t\right)={\left[\widetilde{Y}\left(t\right),\widetilde{Y}\left(t-1\right),\dots ,\widetilde{Y}\left(t-R+1\right)\right]}^{\mathrm{T}},$$

$$\widetilde{{\varvec{s}}}\left(t\right)={\left[s\left(t\right),s\left(t-1\right),\dots ,s\left(t-L-R+2\right)\right]}^{\mathrm{T}},$$

4 $$\widetilde{{\varvec{\varepsilon}}}\left(t\right)={\left[\widetilde{\varepsilon }\left(t\right),\widetilde{\varepsilon }\left(t-1\right),\dots ,\widetilde{\varepsilon }\left(t-R+1\right)\right]}^{\mathrm{T}},$$

where $L$ denotes the twitch rise waveform length and $R$ denotes the extension factor. Then, the linear instantaneous mixture model is defined as:

5 $$\widetilde{{\varvec{Y}}}=\widetilde{{\varvec{A}}}\widetilde{{\varvec{s}}}+\widetilde{{\varvec{\varepsilon}},}$$

where $\widetilde{{\varvec{A}}}$ contains the twitch rise waveforms ${h}_{i}$. To estimate the spike train ${\varvec{s}}=s\left(t\right),t=\{\mathrm{0,1},\dots ,T\}$ using Eq. (5), we used a linear instantaneous BSS model referred to as independent component analysis (ICA) [24].

#### Algorithm

**Fig. 3:**
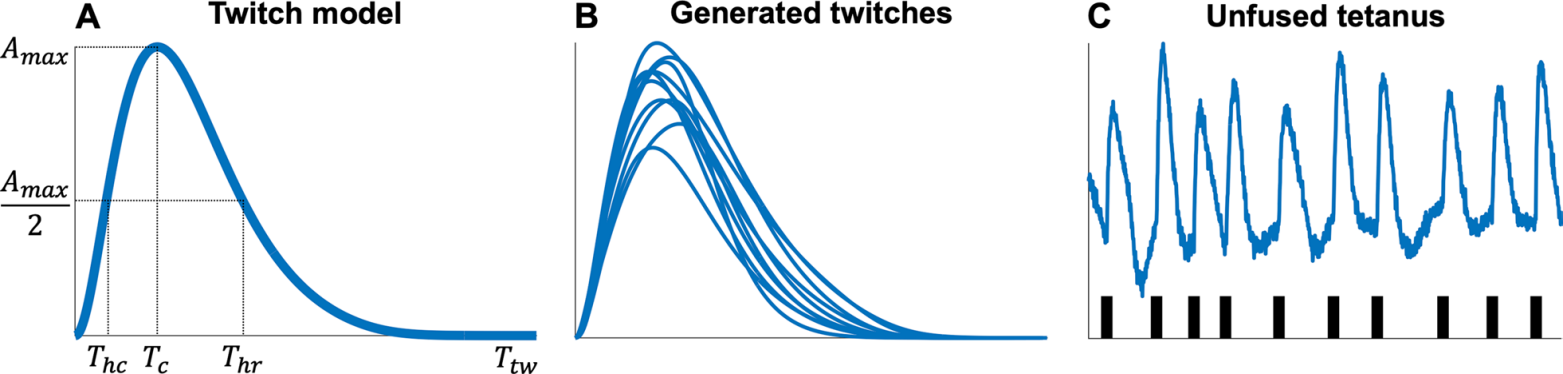
Simulating an unfused tetanic signal using a twitch model and a spike train. **A** A twitch (blue line) is generated by its five parameters (black dashed lines) of the model are the half-contraction time *T*_*hc*_ (ms), the contraction time *T*_*c*_ (ms), the half-relaxation time *T*_*hr*_ (ms), the twitch duration *T*_*tw*_ (ms), and the maximal amplitude *A*_*max*_ (arbitrary unit, a.u.). **B** Generated twitches using the twitch model in **A** by randomly sampling their parameters from a uniform distribution. **C** Time instants (spikes, black vertical lines) for each twitch were simulated using a Gaussian renewal process. The unfused tetanus was obtained by summating the twitches in **B** at their respective time instants with added normally distributed noise to achieve a certain signal-to-noise (SNR) level. Note that the unfused tetanic signal has been differentiated with respect to time, providing unfused tetanus concerning the rate of change (e.g., yank or velocity instead of force or displacement)

Given an unfused tetanic signal (Fig. 3A), the spike train ${\varvec{s}}$ was estimated by solving the separation problem in Eq. (5) using a fixed-point algorithm [25], which has been used for decomposing multichannel EMG signals [2, 3]. After extending the signal (Fig. 3B), the next step, common to most BSS algorithms [24], was to spatially whiten the extended observation matrix using eigenvalue decomposition (Fig. 3C). The whitened extended observation matrix had a covariance matrix equal to the identity (Fig. 3C). After whitening, the fixed-point algorithm estimates the separation (projection) vector and, thereby, the source estimate (Fig. 3D). Here, the separation vector was initialised using a normally distributed random vector. The fixed-point algorithm is based on maximising the non-Gaussian distribution of the source vector using a cost function $G\left(\bullet \right)$ [25]. We used a contrast function associated with sparseness since the spike train is sparse (mostly zeros with a few ones). Here, we choose $G\left(x\right)=\mathrm{log}\left(\mathrm{cosh}\left(x\right)\right)$ due to its kurtotic distribution in line with the characteristics of a spike train that is kurtotic (and skewed) in contrast to a normal distribution.

After obtaining the source estimate, the spike train was estimated by peak detection to identify the time instants of the local maxima. The peak detection was based on (1) the height of the peaks and (2) the distance between consecutive peaks in milliseconds (Fig. 3E). The height of the peaks was based on the number of mean absolute distances ($nMAD$) of the source estimate. The distance between the consecutive peaks was based on the minimum peak distance ($MPD$) where the highest peak within that distance was selected. The resulting time instants of the local maxima were considered the estimated firing times of the spike train ${\varvec{s}}$ (Fig. 3F). The overall CBSS algorithm is summarised in pseudo-code below:
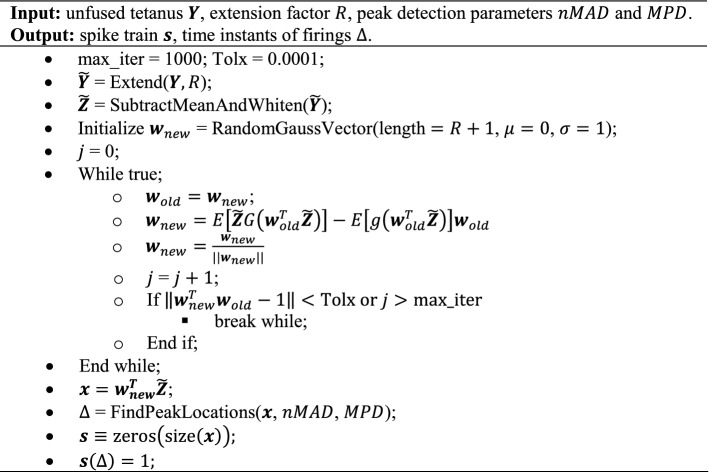


### Parameter evaluation for CBSS

We evaluated the algorithms’ three parameters jointly (based on simulations): the extension factor $R$ and peak detection parameters $nMAD$ and $MPD$. After initial tests to locate the global maxima, we evaluated the following parameters: $R\in \left[\mathrm{10,15},\dots ,40\right]$, $nMAD\in \left[\mathrm{1.0,1.5},\dots ,4.0\right]$, and $MPD\in \left[\mathrm{10,15},\dots ,40\right]$ ms. For each parameter combination, 2700 unfused tetani were generated based on the combinations of SNR values (30, 20, and 10 dB), ISI CV values (5, 20, and 40%), and firing rates (8, 12, and 16 Hz) as explained in a previous study [12].

The rate of agreement (RoA) between the simulated (or reference) and estimated spikes was based on the following metric: $\mathrm{RoA}=\frac{100\times \mathrm{CS}}{\mathrm{CS}+\mathrm{FS}+\mathrm{MS}}$, where $CS$ was the number of correctly identified spikes, $\mathrm{FS}$ was the number of false spikes, and $\mathrm{MS}$ was the number of missed spikes. The tolerance window for correctly identified spikes was set between 0 and $R+10$ (extension factor delay) ms. This tolerance was selected based on maximal RoA (see Additional file 1: Fig. S1). We also analysed the time between a correctly estimated spike ($\mathrm{CS}$) and the simulated spike (referred to as spike delta) and its variability (spike delta variability) for different extension factors $R$. The difference in spike delta variation between different extension factors (in parameter evaluation) was tested using Levene’s test with a significance level of 5% [26].

### Comparison between CBSS and HWM—simulated and experimental data

The parameters that maximised the mean RoA of CBSS were extracted based on averaging the RoA of all simulated parameter combinations. For HWM, we used the optimised parameters from a previous study with a similar parameter evaluation procedure using the same RoA threshold (between − 5 and 25) [12]. Note that HWM consists of three steps: (1) computing the continuous Haar wavelet transform of the signal at a given pseudo-frequency, (2) standardising the wavelet transform coefficients based on z-score, and (3) finding the time instants of the local maxima of the wavelet coefficients using peak detection [12].

We reported the mean and standard deviation of the RoA values for each method, and we tested the pairwise differences between the spike trains of CBSS and HWM in three ways. First, we compared them using the same simulation procedure in the parameter evaluation for various SNR, ISI CV, and firing rate values and tested the pairwise differences using Wilcoxon signed rank test. Second, we compared them using the experimental unfused tetani. Third, we calculated the computational time to run each algorithm on the simulated data (2700 times) and tested the pairwise differences between the algorithms. The significance level was set to 5%, and the *p*-values were corrected using Bonferroni correction.

## Results

### Parameter evaluation for CBSS—simulations

**Fig. 4:**
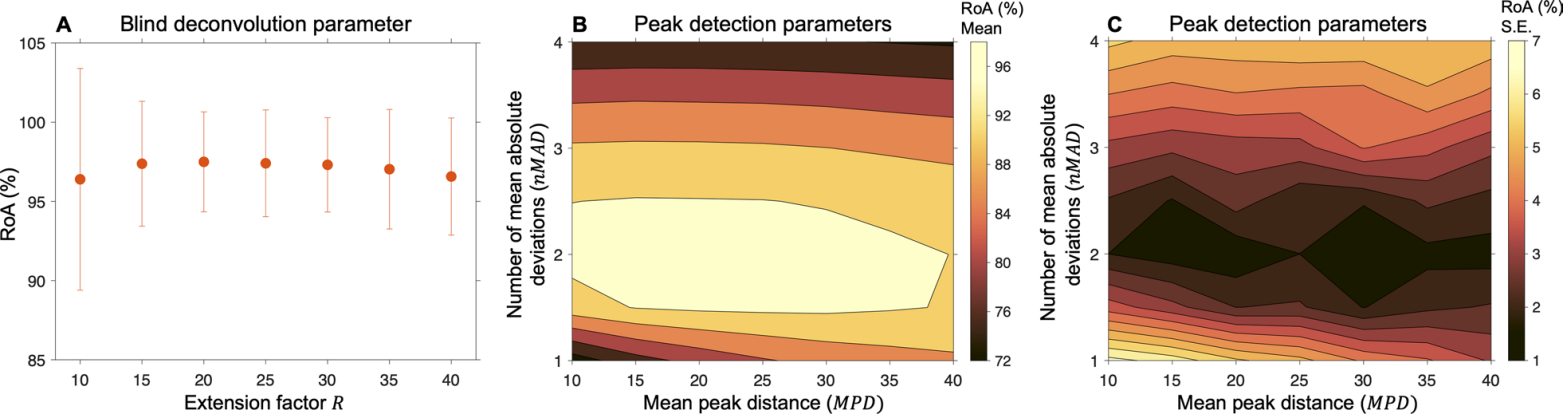
Parameter evaluation of the convolutive blind source separation (CBSS) algorithm using many simulations. **A** The mean rate of agreement (RoA) and its 95% standard error for different extension factors $R$. Highest mean RoA was for $R=20$. **B** Given the extension factor $R=20$, the peak detection parameters ($n\mathrm{MAD}$ and $\mathrm{MPD}$) that had the highest mean RoA were $n\mathrm{MAD}=2$ and $\mathrm{MPD}=20$. **C** The standard errors associated with the mean RoA values in **B**

The highest mean RoA for CBSS was achieved when $R=20$ with 97.5 ± 1.6% (Fig. 4A). Given extension factor $R=20$, we found that the peak parameters associated with the highest RoA values and smallest standard errors were $n\mathrm{MAD}=2$ and $\mathrm{MPD}=20$ (Fig. 4B, C).

**Fig. 5:**
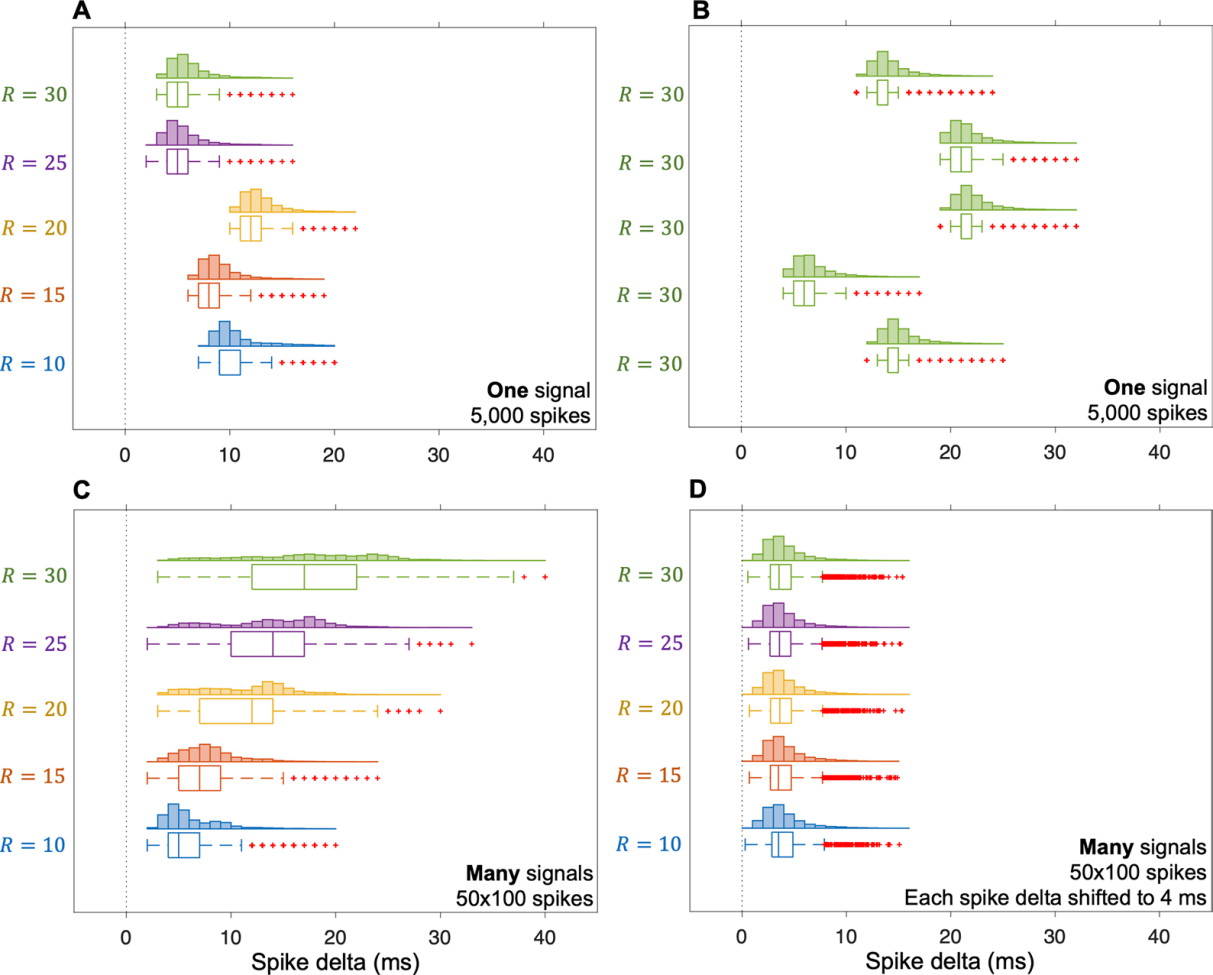
Spike delta and spike delta variability for different extension factors ($R$) regarding CBSS. **A** The spike delta differed for different $R$, but there was no difference in spike delta variability. **B** The spike delta differed for the same $R$ for five reiterations of the algorithm, but there was no difference in spike delta variability. **C** The spike delta differed for different $R$, and there was a difference in spike delta variability. **D** Subtracting the spike delta for each simulated signal: the spike delta did not differ for different $R$, and there was no difference in spike delta variability

We found that the spike delta differed for different extension factors (Fig. 5A), but also for the same extension factor at different iterations (Fig. 5B). The larger the extension factor, the greater variability in the mean spike delta (Fig. 5C). However, there was no difference (*p* > 0.05) in spike delta *variability* for the different extension factors (Fig. 5A, B, D). The spike delta variation was 2.2 ± 0.5 ms.

### Comparison between CBSS and HWM—simulated data

Considering all parameter combinations, CBSS had a higher RoA than HWM (97.5 ± 2.7 vs 96.9 ± 3.3, *p* < 0.001). By considering each simulation parameter separately, CBSS had a higher RoA than HWM in 12 of the 27 simulation parameter combinations (Table 1). In contrast, HWM had a higher RoA than CBSS in 6 parameter combinations. For the other nine combinations, there was no difference in RoA. Finally, CBSS was more robust to high ISI CV values than HWM (Table 1). However, there was no clear pattern with respect to noise levels or firing rates.

**Table 1:** Comparing the performance of CBSS and HWM to estimate spikes based on simulated signals with varying parameters

Simulation parameters			CBSS	HWM	Pairwise difference	*p*-value
FR (Hz)	ISI CV (%)	SNR (dB)	RoA (%)	RoA (%)		
8	5	30	99.3 ± 0.8	**100.0 ± 0.2**	− 0.7 ± 0.8	***
8	5	20	99.7 ± 0.5	99.9 ± 0.3	− 0.2 ± 0.6	0.21
8	5	10	99.7 ± 0.6	99.5 ± 0.7	0.2 ± 1.0	1.00
8	20	30	99.1 ± 0.8	**99.6 ± 0.7**	− 0.4 ± 1.1	*
8	20	20	99.5 ± 0.8	99.5 ± 0.7	0.0 ± 0.9	1.00
8	20	10	**99.2 ± 0.9**	98.0 ± 1.3	1.3 ± 1.4	***
8	40	30	**97.5 ± 1.6**	97.3 ± 2.0	0.3 ± 1.6	*
8	40	20	**98.0 ± 1.4**	96.8 ± 1.7	1.3 ± 1.8	***
8	40	10	**97.2 ± 1.7**	93.9 ± 2.5	3.2 ± 2.3	***
12	5	30	98.8 ± 0.5	98.7 ± 1.1	0.1 ± 1.3	0.38
12	5	20	98.8 ± 0.5	98.9 ± 1.0	− 0.1 ± 1.0	1.00
12	5	10	98.0 ± 1.2	**98.8 ± 1.0**	− 1.0 ± 1.4	***
12	20	30	**98.8 ± 0.6**	98.5 ± 1.2	0.4 ± 1.5	**
12	20	20	**98.9 ± 0.7**	98.4 ± 1.4	0.3 ± 1.1	***
12	20	10	97.6 ± 1.7	**98.6 ± 1.2**	− 0.8 ± 1.5	*
12	40	30	**97.3 ± 1.5**	95.2 ± 2.4	2.4 ± 2.2	***
12	40	20	**97.4 ± 1.6**	95.3 ± 2.6	1.9 ± 2.4	***
12	40	10	95.2 ± 2.6	94.9 ± 2.0	0.4 ± 2.5	1.00
16	5	30	98.4 ± 1.1	98.4 ± 1.2	0.1 ± 1.6	1.00
16	5	20	98.3 ± 0.8	98.4 ± 1.2	− 0.2 ± 1.4	1.00
16	5	10	94.8 ± 2.3	**98.3 ± 1.3**	− 3.8 ± 1.8	***
16	20	30	**98.5 ± 1.0**	96.8 ± 1.4	2.0 ± 2.1	***
16	20	20	**98.1 ± 1.5**	96.7 ± 1.8	1.6 ± 1.7	***
16	20	10	93.6 ± 2.3	**96.4 ± 1.7**	− 3.3 ± 2.4	***
16	40	30	**96.0 ± 2.2**	90.4 ± 2.8	5.6 ± 3.6	***
16	40	20	**95.6 ± 2.0**	90.4 ± 2.6	5.1 ± 3.5	***
16	40	10	89.6 ± 4.3	90.6 ± 3.2	− 0.6 ± 3.5	1.00
Mean ± SD (across all parameter combinations)			97.5 ± 2.7	96.9 ± 3.3	0.6 ± 2.8	***

*FR* firing rate, *ISI* inter-spike interval, *CV* coefficient of variation, *SNR* signal-to-noise ratio, CBSS = convolutive blind source separation, *HWM* Haar wavelet method, *RoA* rate of agreement. *p < 0.05, **p < 0.01, ***p < 0.001 using Bonferroni correction for multiple comparisons

**Fig. 6:**
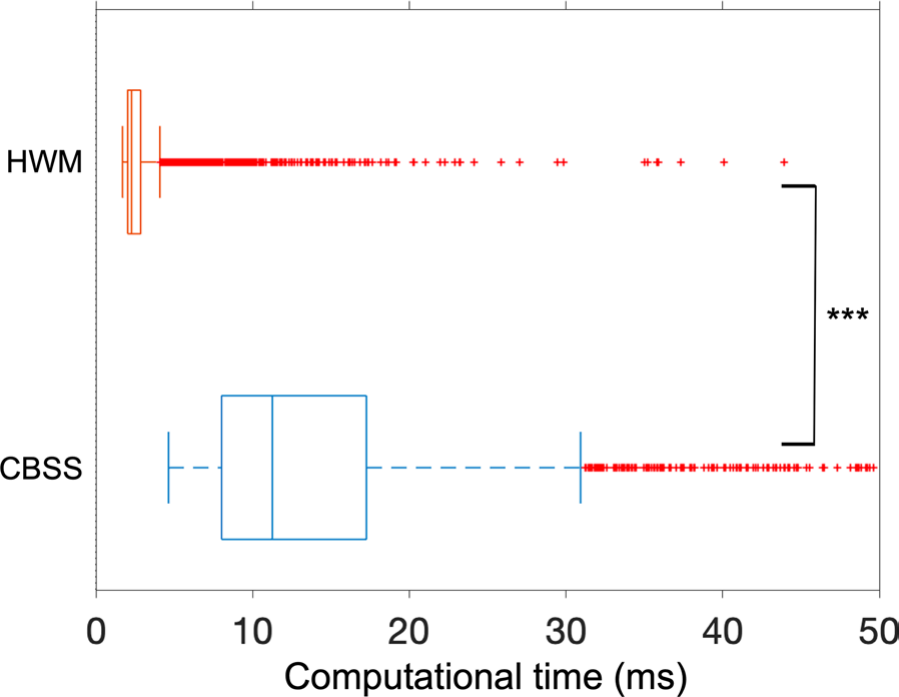
The computational time for the convolutive blind source separation (CBSS) and the Haar wavelet method (HWM). Each boxplot is based on 2700 computational time values, i.e. 100 signals containing 100 spikes for 27 different simulation parameters. ***p < 0.001

The computational time was much lower for HWM than for CBSS (*p* < 0.001). However, there is no practical difference since the computational time of HWM is approximately 2 ms and CBSS 11 ms for a signal that is about 10 s (Fig. 6).

### Comparison between CBSS and HWM—experimental data

The three unfused tetani from human biceps brachii had an average firing rate of 11.4 Hz and an average ISI CV of 13.4%. The estimated spike trains from CBSS and HWM highly agreed with the EMG reference spikes (98.0 ± 3.4% and 96.3 ± 3.2%, respectively). See Table 2.

**Table 2:** Comparing the performance of CBSS and HWM to estimate spikes based on experimental signals (voluntary and evoked contractions)

Voluntary contractions		Evoked contractions		CBSS	HWM	Pairwise	*p*-value
Unfused tetani	Spikes	Unfused tetani	Spikes	RoA (%)	RoA (%)	difference	
3	58			98.0 ± 3.4	96.3 ± 3.2	1.7 ± 3.3	#
		18	720	98.0 ± 2.8	96.5 ± 5.6	1.1 ± 4.5	0.24

*CBSS* convolutive blind source separation, *HWM* Haar wavelet method, *RoA* rate of agreement (between estimated and EMG reference spikes). # To few unfused tetani to do a statistical test

The 18 unfused tetani from rat gastrocnemius had an average firing rate of 14.4 Hz and an average ISI CV of 30.6%. The estimated spike trains from CBSS and HWM highly agreed with the EMG reference spikes (98.0 ± 2.8% and 96.5 ± 5.6%, respectively). Given this, there was no difference between the two methods (*p* = 0.24). See Table 2.

## Discussion

This work aimed to estimate simulated and experimental unfused tetani spike trains using CBSS and compare it against HWM [12]. For this purpose, we evaluated the parameters of CBSS using simulations and explored the algorithm’s parameters’ relation with the spike delta and its variation. Then, we compared CBSS against the HWM based on their RoA using simulated and experimental unfused tetanic signals from voluntary contractions of humans and evoked contraction of rats. The main finding was that CBSS had (on average) a higher performance than HWM with respect to the simulated firings than HWM (97.5 ± 2.7 vs 96.9 ± 3.3, *p* < 0.001). In addition, we found that the estimated spike trains from CBSS and HWM highly agreed with the experimental spike trains (98.0% and 96.4%).

We have shown that CBSS can be used to estimate the spike train of an unfused tetanic signal since the firings highly agree with the simulated (see Fig. 4A and Table 1) and EMG reference spike trains from two different experimental datasets (see Table 2). We found that the spike delta variability did not depend on the extension factor, although the spike delta differed for different extension factors and the same extension factor (Fig. 5A, B). This observation suggests that there are multiple local maxima, and the convergence to different local maxima may depend on the initialisation of the separation vector [2]. Also, a larger extension factor leads to a larger spike delta variance (between trials, Fig. 5C), suggesting that the convergence issue increase with the extension factor value. This problem could be overcome using a more standardised initiation of the separation factor or finding a standardised way to shift the estimated spikes without knowing the ground truth. Although there is potential for improvement, both CBSS and HWM used optimal RoA thresholds of the same length (0 to 30 ms and − 5 to 25 ms, respectively), where CBSS had, on average, better performance than HWM (Table 1).

The computational time was about five times slower for CBSS than for HWM. This finding is explained by CBSS extending the signal from a vector to a matrix to make the convolutive approach an instantaneous linear problem suitable for independent component analysis (ICA) [2, 3]. Then, fixed-point iterations are used to find a projection vector. However, this computational time difference is of no practical difference since the computational time of HWM is approximately 2 ms and CBSS 11 ms for a signal that is about 10 s (Fig. 6). For example, a time difference of 200 ms between visual feedback and movement leads to the perception of a delay [27]. The computational times are well within that time difference without optimisation.

This study considered observations of unfused tetani. Although CBSS can be used directly to estimate firings based on the estimated unfused tetanus from the ultrasound-based pipeline [6, 12], this study indicates the potential of either extending or including the temporal CBSS approach to the current spatially focused BSS method [6, 28] to improve the separation of displacement velocity images from ultrasound to increase the identification rate [8]. Another solution would bypass the estimation of an unfused tetanic signal (Fig. 1D) and go directly to spikes (from Fig. 1C to Fig. 1E), thereby reducing the pipeline’s exposure to error propagation, i.e. estimating spikes based on occasionally poor estimates of an unfused tetanic signal. Given this, the next challenge emerges when expanding to ultrasound images, i.e. successive twitches within the same MU may differ. There is a possibility that twitches from a MU may be highly similar to the ones of other MUs. However, one could overcome this challenge by including spatial information, which has a high resolution (< 1 mm) as the MU relates to a physical component (muscle unit) in the spatial domain. A potential solution may be expanding current sEMG decomposition algorithms [2, 3] to include spatial dependence or sparsity in addition to the temporal deconvolution and validating it using an authentic simulation model [29]. Yet, all these approaches need to deal with the motion of non-MU-related structures that hides a large part of the movement caused by a MU in ultrasound images [11]. One potential solution could be to use the spatiotemporal clutter filtering approach to improve the sensitivity to detect microvascular networks or blood flows corrupted by significant tissue or probe motion artefacts [30]. Nevertheless, the implementation and validation of these approaches will be investigated in the future.

In conclusion, this study estimated simulated and experimental unfused tetani spike trains using a CBSS algorithm and compared its performance against a previously optimised method, i.e. HWM. We found that the estimated spike trains from CBSS and HWM highly agreed with the simulated and EMG reference spike trains, and CBSS had, on average, higher performance. This result implies that the CBSS of an unfused tetanic signal can be used to estimate its spike train, and it can be used directly within the current ultrasound-based MU identification pipeline. Extending this approach to decomposing ultrasound images into spike trains directly is promising. However, it remains to be investigated in future studies where spatial information is inevitable as a discriminating factor.

## Supplementary Information

**Additional file 1. Table S1.** The simulation parameters. **Figure S1.** Sensitivity analysis of the tolerance interval for rate of agreement (RoA) calculation using different values of extension factor $R$ (10, 20, and 30). We considered a simulation of 5,000 spikes and subsequent twitches using a firing rate of 12 Hz, an ISI CV of 20%, and an SNR of 20 dB. The red crosses denote the selected tolerance intervals.

## Data Availability

The datasets supporting the conclusions of this article are included within the article (and its additional file). The code and data are available on request from the corresponding author RR. For requests about the experimental dataset 2 (on rats), the corresponding author RR will pass the request to Professor Jan Celichowski.
